# Evaluation of the Functional and Radiological Outcomes of Antibiotic-Coated Intramedullary Interlocking Nailing for Gustilo and Anderson Grades 2 and 3 Open Tibial Shaft Fractures

**DOI:** 10.7759/cureus.22357

**Published:** 2022-02-18

**Authors:** Darshan Patel, Nagakumar J S, Sandesh Agarawal, Amith Kamath

**Affiliations:** 1 Department of Orthopaedics, Sri Devaraj Urs Academy of Higher Education and Research, Kolar, IND

**Keywords:** gustilo and anderson classification, open tibial fracture, infection, intramedullary nail, antibiotic coated

## Abstract

Background: Tibial shaft fractures are the most common fractures among long bones. At present, implants coated with broad-spectrum antibiotics have been developed, and antimicrobial eluting implants are widely used in clinical practice.

Materials and methods: This prospective study was conducted among 40 patients with tibial shaft fractures who visited the Department of Orthopedics in RL Jalappa Hospital, Tamaka, Kolar, Karnataka, from February 2021 to September 2021. As it is a large trauma center near the national highway, all 40 cases, including the referral cases, were operated within two months of the initiation of the study, with the last case operated in March 2021. The inclusion criteria were: patients aged more than 18 years, diaphyseal tibial fractures definitively treated by antibiotic coated intramedullary nailing, and Gustilo and Anderson grades 2, 3A, and 3B open tibial shaft fractures. All patients with grades 2, 3A, and 3B open fractures of the tibial shaft were treated with antibiotic-coated nails and followed up at one, three, and six months post-surgery.

Results: The mean age of patients was 35.6 years, and the mean union time of fractures was 4.2 months. Road traffic accidents (RTA) are the most common etiology for tibial bone fractures. In this study, grade 3A open fractures had the highest number of cases (N = 26). No patients in the present study developed superficial or deep infections post-operatively. All patients were assessed with Johner-Wruhs criteria at each follow-up, and they showed improvement in knee and ankle joint mobility, pain, and deformity. Most patients achieved good functional results after postoperative follow-up, followed by those with excellent results. According to the radiographic union scale in tibial shaft fractures criteria, 23 patients showed good radiological results after postoperative follow-up, followed by 15 patients with excellent and 2 patients with fair results.

Conclusion: Most of the patients showed good to excellent functional and radiological results according to Johner-Wruhs and Radiographic Union Scale for Tibial fractures (RUST) criteria. The use of antibiotic-coated nails to treat compound tibial fractures was associated with a decreased risk of deep wound infections and good fracture healing.

## Introduction

Long bone fractures are crippling injuries that generally result from high-energy trauma. With the mechanization of mobility, rapid industrialization, and urbanization, the number of vehicles and vehicular traffic has greatly increased, which has increased the incidence of vehicular accidents [1].

Tibial shaft fractures are the most common fractures among long bones. Among tibial fractures, half are open injuries, which increases vulnerability to infection [2-5]. According to the Gustilo and Anderson classification system, the probability of infection in compound fractures increases with the severity of the injury, from <2% in grade 1 to 50% in grade 3 open fractures [6].

Different types of treatment for open fractures are available, including two-staged treatment with external fixation, which is the gold standard for open fractures, plaster immobilization, debridement, and surgical stabilization. Recognizing the extent of involvement of soft tissue along with the structure of the fracture is very important in the selection of proper treatment approaches [7]. However, despite advances in surgical techniques and antibiotics, wound infection and osteomyelitis can still occur. In Gustilo and Anderson grade 3 open fractures, the probability of deep infections is around 80% [8].

Several studies concluded that antibiotic treatment decreases the probability of infection in open fractures. Implants coated with broad-spectrum antibiotics have been developed, and antimicrobial eluting implants are widely used in clinical practice [9,10]. According to a study on the resource and cost-effectiveness of using antibiotic-coated nails compared with uncoated nails in open tibial fractures at four European centers, antibiotic-coated intramedullary nails were more cost-saving in patients with a higher risk of infection (Gustilo and Anderson type 3) [11]. The present study was conducted to know the functional and radiological outcomes of antibiotic-coated intramedullary interlocking nails for compound tibial shaft fractures using Johner-Wruhs and Radiographic Union Scale for Tibial fractures (RUST) criteria, respectively.

## Materials and methods

This prospective study was conducted among 40 patients with tibial shaft fractures who visited the Department of Orthopedics in RL Jalappa Hospital, Tamaka, Kolar, Karnataka, from February 2021 to September 2021. As it is a large trauma center near the national highway, all 40 cases, including the referral cases, were operated within two months of the initiation of the study, with the last case of the study operated in the month of March 2021. Data collection was performed after obtaining ethical approval from the institutional ethics committee of Sri Devaraj Urs Medical College (approval number DMC/KLR/IEC/422/2021-22) and informed consent from study subjects. The inclusion criteria were: patients aged more than 18 years, diaphyseal tibial fractures definitively treated by antibiotic-coated intramedullary nailing within 24 hours of presentation, and Gustilo and Anderson grades 2, 3A, and 3B open shaft of tibia fractures. The exclusion criteria were as follows: associated neurovascular injury, compartment syndrome, and allergy to aminoglycosides.

All patients with grades 2, 3A, and 3B open tibial fractures were treated with antibiotic-coated nails. The intramedullary nails used in this study were made of stainless steel or titanium. According to the antibiotic policy of our hospital, a combination of gentamicin and vancomycin amounting to 50 mg each in 80 gm of polymethylmethacrylate (PMMA) cement (Palacos R + G bone cement) was used to coat the nail using the silicon tubing technique for all patients. The preparation of the antibiotic-coated nail was done in the operation theatre. The medullary canal of the bone was reamed 2 mm wider than the antibiotic-coated nail diameter, as 1 mm of coating is provided by the cement around the nail. Thus, with reaming, the antibiotic-coated nail was easily placed without dislodging the cement. Following nail implantation, vancomycin was sprayed over the open wound, assumably to reduce the chances of superficial wound infections. Patients were followed up at one, three, and six months post-surgery to assess functional and radiological outcomes, which were evaluated on the basis of the Johner-Wruhs and RUST criteria, respectively.

A general and systemic examination was conducted as per protocols. The data were recorded on an Excel sheet. A descriptive analysis was performed, and the results are presented in the tables.

## Results

Our study showed that 4 (10%), 18 (45%), 10 (25%), and 8 (20%) patients belonged to the 20-30-, 30-40-, 40-50-, and 50-60-year-old age groups, respectively. The mean age of patients was 35.6 years (SD 6.8). 32 (80%) of the patients were males, and eight (20%) were females. A total of 35 (87.5%) and 5 (12.5%) cases were admitted due to road traffic accidents (RTA) and fall from height, respectively. In the present study, 15 patients achieved fracture union at 3.5 months, 23 patients at 4.5 months, and 2 patients at 6 months, with a mean union time of 4.2 months (SD 1). No patients in the present study developed superficial or deep infections post-operatively. According to the Gustilo and Anderson classification system, 9 (22.5%), 26 (65%), and 5 (12.5%) patients had type 2, 3A, and 3B fractures, respectively. The patients' characteristics are summarized in Table 1.

**Table 1:** Characteristics of patients RTA, road traffic accident; SD, standard deviation; GA, Gustilo–Anderson

Age group (in year)	Number (%)
20–30	4 [10.0]
30–40	18 [45.0]
40–50	10 [25.0]
50–60	8 [20.0]
Mean ± SD	35.6 ± 6.8 years
Gender	
Male	32 [80.0]
Female	8 [20.0]
Mode of injury	
RTA	35 [87.5]
Fall from height	5 [12.5]
Mean union time (mean ± SD)	4.2 ± 1.0 months
Fracture type (GA classification)	
2	9 [22.5]
3A	26 [65.0]
3B	5 [12.5]

According to the Johner-Wruhs criteria, 1 (2.5%), 22 (55%), and 17 (42.5%) patients showed fair, good, and excellent improvement at six months post-surgery, respectively (Table 2).

**Table 2:** Johner-Wruhs criteria at one, three, and six months of post-operative follow-up [N=40]

Duration	Johner–Wruhs criteria			
	Excellent	Good	Moderate	Poor
1 month post-operatively	13 [32.5]	23 [57.5]	4 [10.0]	0 [0.0]
3 month post-operatively	15 [37.5]	24 [60.0]	1 [2.5]	0 [0.0]
6 month post-operatively	17 [42.5]	22 [55.0]	1 [2.5]	0 [0.0]

**Figure 1:**
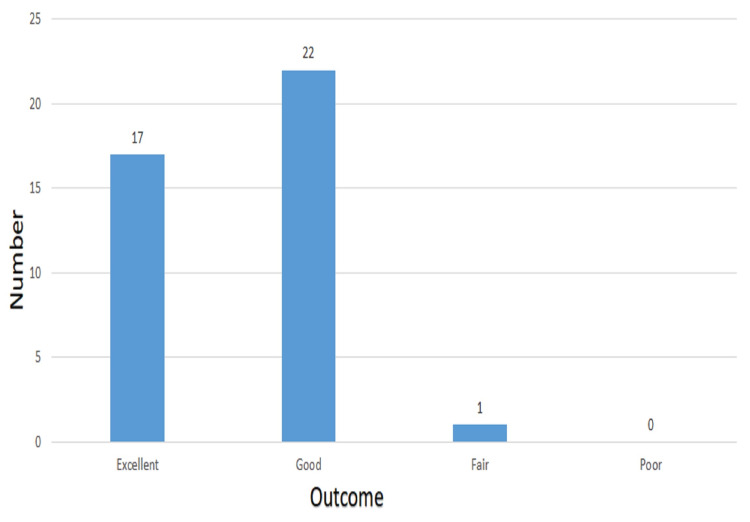
Functional outcome at six months of postoperative follow-up [N = 40]

Figure 1 shows that 1 (2.5%), 17 (42.5%), and 22 (55%) patients had fair, excellent, and good functional outcomes at six months of postoperative follow-up, respectively.

**Figure 2:**
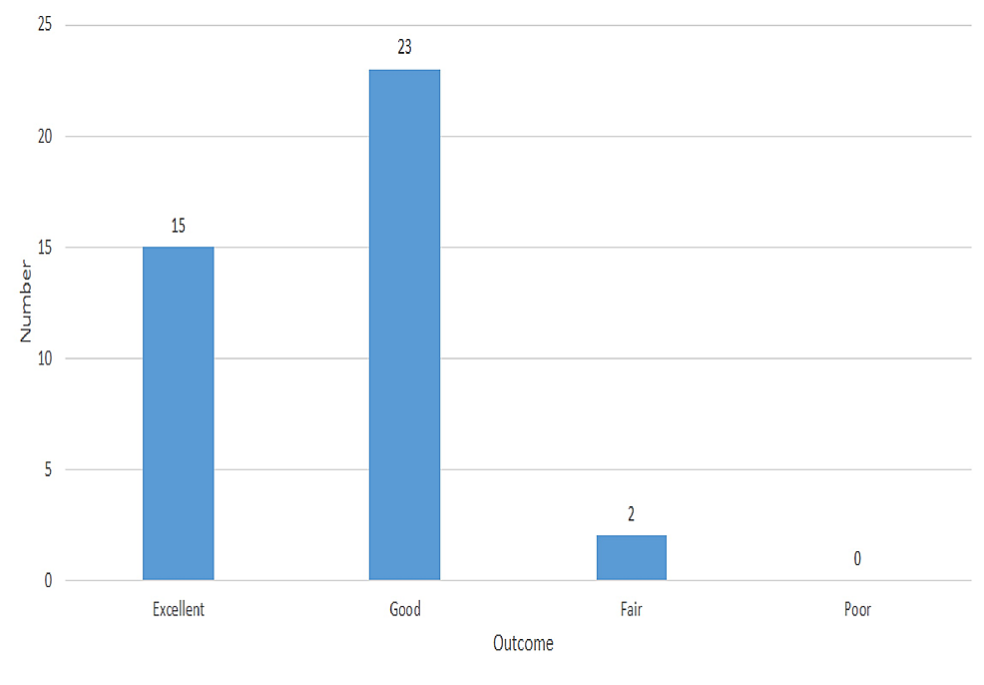
Radiological outcome according to the RUST criteria at six months of postoperative follow-up [N = 40]

Figure 2 shows that 15 (37.5%), 23 (57.5%), and two (5%) patients had excellent, good, and fair radiological outcomes, respectively, at six months of postoperative follow-up according to RUST criteria.

## Discussion

Infected fractures of the long bone require interventions to achieve union, infection control, and to provide stability. Delivery of antibiotics locally and systemically and surgical debridement are used to control the infection [12]. Infection control is very important and difficult to achieve in orthopedics. Occasionally, it may not be possible to completely remove the probability of infection in compound fractures [13].

In the present study, the highest number of patients belonged to the 30-40-year-old age group, followed by the 40-50-year-old age group. The mean age of patients was 35.6 years. These findings correlate with those of Khan et al. [14], Salem [15], Pratap et al. [16], Uikey et al. [1], and Vignesh et al. [17]. The male/female ratio was 4:1, which is similar to that observed by Vignesh and Ghai [17], Zhang et al. [18], Uikey et al. [1], Lin and Hou [19], Salem [15], Court-Brown et al. [3], and Prakash et al. [20]. The present study found that RTA was the most common cause of tibial bone fracture. A similar finding was also observed by Prakash et al. [20], Court-Brown et al. [3], Pratap et al. [16], Uikey et al. [1], Vignesh et al. [17], and Zhang et al. [18].

Vancomycin and gentamicin are preferred for local delivery due to their properties of heat stability and wide-spectrum antibiotic activity. Elution of high concentrations of antibiotics from bone cement does not cause systemic side effects [12].

In the present study, 15 patients achieved fracture union at 3.5 months, 23 patients at 4.5 months, and 2 patients at 6 months, with a mean union time of 4.2 months, whereas the mean union time in a study conducted by Pratap et al. [16] was 16.5 weeks. In the study by Prakash et al., fracture union was achieved in 29 (96.67%) out of 30 patients, and non-union was observed in only one patient (3.33%) [20]. The present study was in accordance with the studies of Pratap et al. [16] and Fuchs et al. [21], where none of the patients underwent non-union.

In the present study, no patient developed a superficial or deep infection post-operatively, while in the study conducted by Pratap et al. [16], two patients developed superficial infections without any deep infection.

In the present study, most patients had grade 3A fractures according to the Gustilo and Anderson classification system. In the study by Prakash et al., 15 (50%), 13 (43.33%), and two patients (6.67%) had grades 2, 2A, and 3B compound fractures, respectively [20]. In the study by Prakash et al., 13 (52%) of the cases had grade 2 fractures, whereas 12 (48%) of the cases had other types of fractures [16]. Salem found that eight (72.72%) patients had Gustilo and Anderson grade 2 fractures, whereas three (27.27%) patients had grade 3A fractures [15].

In the present study, according to Johner-Whrus criteria, 22 (55%) patients had a good functional outcome, followed by 17 (42.5%) patients with an excellent outcome, and only one (2.5%) patient had a fair outcome at six-month follow-up, while in the study conducted by Reddy Konda et al. [22], in which 39 (86.7%) out of 45 patients had an excellent functional outcome, 5 (11.1%) patients had a good and 1 (2.2%) had a fair outcome. One patient (2.5%) who had a fair outcome was due to reduced knee joint mobility and a 7-degree valgus deformity at six-month follow-up. The present study used the RUST criteria to evaluate the radiological outcomes. According to the criteria, 23 (57.5%) patients showed good radiological results after postoperative follow-up, followed by 15 (37.5%) with excellent results. Only two (5%) patients had a fair outcome as radiologically two cortices were united, with bridging callus formation over the third cortex. In Prakash P et al.’s study, 3 (10%), 24 (80%), and 3 (10%) patients had excellent, good-to-fair, and poor outcomes, respectively [20].

Limitations

The present study has some limitations, including the relatively smaller sample size and brief follow-up period. Moreover, various potential factors that influence the functional and radiological outcomes of tibial shaft fractures, including sedentary lifestyle, occupation, smoking, alcohol consumption, diabetes mellitus, and chronic drug intake (e.g., NSAIDS [non-steroidal anti-inflammatory drugs] and steroids), were not investigated.

## Conclusions

The present study showed that most of the patients exhibited good to excellent functional and radiological results according to Johner-Wruhs and RUST criteria, respectively, after treatment with antibiotic-coated nails. The use of antibiotic-coated nails to treat compound tibial fractures was associated with a decreased risk of deep wound infections and good fracture healing. The antibiotic-coated intramedullary interlocking nail is a good treatment option for compound tibial fractures, yielding good functional outcomes in open tibial fractures with fewer complications, and should be used whenever indicated.
